# Changes in Quality of Life, Depression, and Menopausal Symptoms After Surgical Menopause and the Efficacy of Hormone Replacement Therapy in Gynecological Cancer Survivors: A One-Year Prospective Longitudinal Study

**DOI:** 10.3390/medicina61071191

**Published:** 2025-06-30

**Authors:** Noriko Karakida, Shintaro Yanazume, Natsuko Uchida, Mika Sakihama, Tsutomu Douchi, Hiroaki Kobayashi

**Affiliations:** Department of Obstetrics and Gynecology, Kagoshima University School of Medicine, Kagoshima 890-0075, Japan

**Keywords:** gynecological cancer survivors, quality of life, surgical menopause, menopausal symptoms, depression, hormone replacement therapy

## Abstract

*Background and Objectives*: This study investigated changes in quality of life (QOL), depression, and menopausal symptoms after surgical menopause, and the efficacy of hormone replacement therapy (HRT) in gynecological cancer survivors (GCS). *Materials and Methods*: Participants undergoing gynecologic cancer surgery (N = 155) were divided into those who received HRT after surgical menopause (SH, N = 47), those after surgical menopause (SM, N = 54), and those after natural menopause (NM, N = 54). QOL, depression, and menopausal symptoms were assessed using the Functional Assessment of Cancer Therapy-General (FACT-G), Center for Epidemiologic Studies Depression Scale (CES-D), and Endocrine Symptoms Subscale-19 (ESS-19), respectively. Assessments were conducted before and at 6 and 12 months after surgery. *Results*: In SH and SM, FACT-G and CES-D were worst before surgery, gradually improved by 6 months, and remained stable for the following 6 months. FACT-G and CES-D showed an inverse relationship. ESS-19 did not change in SH and SM for 12 months. Among the items on the ESS-19, worsened vasomotor symptoms (VMSs), assessed with ES1, showed more improvement in SH than in SM, while worsened arthralgia assessed with BRM1 was maintained in SM. Multivariate analysis showed that HRT was not independently correlated with changes in QOL and depression status. *Conclusions*: In GCS, the prevalence of depression was highest at cancer disclosure along with declining QOL. QOL gradually improved by 6 months after surgery in SH and SM, but not in NM. Although menopausal HRT is known to alleviate VMS, anxiety, and depression, its efficacy for cancer-related emotional distress and the associated decline in QOL seems limited.

## 1. Introduction

Advances in medicine have led to cancer patients living longer. Meanwhile, similar to the global trend [1,2], endometrial and ovarian cancers are also increasing in Japan. Several risk factors, including late menopausal age, abnormal uterine bleeding, obesity, diabetes, and hypertension, are known to contribute to disease onset and progression [3]. Recent advances, including early detection through ultrasound and biomarkers, surgical improvements, and adjuvant therapies such as chemotherapy, radiotherapy, and hormonal therapy, have improved survival rates [4]. Thus, it is increasingly important to both focus on cancer treatment and to practice medical care aimed at improving the mental health and quality of life (QOL) of gynecological cancer survivors (GCSs). We define “cancer survivors” in accordance with the National Cancer Institute (NCI) as individuals from the time of cancer diagnosis through the balance of life. Gynecological cancers often require the removal of the uterus and ovaries, resulting in a loss of identity in women. Surgical menopause also influences mental health. Compared to natural menopause, surgical menopause induces more rapid, frequent, severe, and long-lasting menopausal symptoms [5,6,7], along with emotional distress, including anxiety and depression, in addition to causing early menopause [8,9]. Recent studies have highlighted that post-treatment sexual dysfunction significantly affects QOL in GCSs. Cianci et al. (2023) reported that gynecological cancer survivors experience persistent sexual dysfunction due to a combination of hormonal, anatomical, and psychological changes following treatment [10]. This includes decreased libido, dyspareunia, and emotional detachment, often overlooked during follow-up care. Similarly, many survivors report diminished sexual satisfaction and greater psychological distress even long after treatment, underlining the need for comprehensive survivorship care that addresses intimate relationships and sexual health [11]. Despite growing evidence, in Japan and other Asian countries, the evaluation of sexual function and emotional well-being remains limited in clinical practice due to cultural barriers and lack of awareness. Therefore, longitudinal studies assessing mental health, menopausal symptoms, and QOL, including sexual function, are urgently needed to support more holistic care for GCSs. Menopausal hormone replacement therapy (HRT) improves QOL by alleviating vasomotor symptoms (VMS), genitourinary syndrome, anxiety, and depression [12,13]. With the exception of patients with breast cancer, recent evidence indicates the efficacy of HRT for GCSs [14,15]. However, most studies regarding HRT address the risk of cancer recurrence, and studies on the efficacy of HRT for menopausal symptoms and QOL in GCSs are limited.

In this study, we conducted a one-year prospective longitudinal study on the changes in QOL, depression, and menopausal symptoms after surgical menopause, and the efficacy of HRT for GCSs.

## 2. Material and Methods

### 2.1. Study Population

Two hundred and four Japanese GCSs were recruited preoperatively between June 2017 and November 2022 at Kagoshima University Hospital. Eligibility criteria included being ≥18 y, being able to read and write in Japanese, and having a diagnosis of one of the following gynecological cancers: endometrial, cervical, or ovarian. Patients with a history of other malignancies or comorbid psychiatric conditions were excluded from the study. After excluding participants in accordance with exclusion criteria, participants were divided into the following three groups: those who received HRT after surgical menopause (SH), those after surgical menopause (SM), and those after natural menopause (NM). Natural menopause was defined as the absence of menstruation for 12 months or more. Surgical menopause was defined as the surgical removal of both ovaries in women who have not yet reached natural menopause. The patients were responsible for making the decision regarding whether to receive HRT following a comprehensive explanation of the study. To investigate changes in QOL, depression, and menopausal symptoms associated with surgical menopause, HRT was started 6 months after surgery. The reason for starting HRT 6 months after surgery is that hot flash symptoms in surgical menopausal patients significantly increase 6 months after surgery [16], and because the only RCT of HRT after uterine cancer surgery included enrollments up to 24 weeks after surgery [17], we decided to start HRT 6 months after surgery. To examine the efficacy of HRT, the SH group filled out a 2nd questionnaire before starting HRT and a 3rd questionnaire after continuing HRT for 6 months.

**Figure 1:**
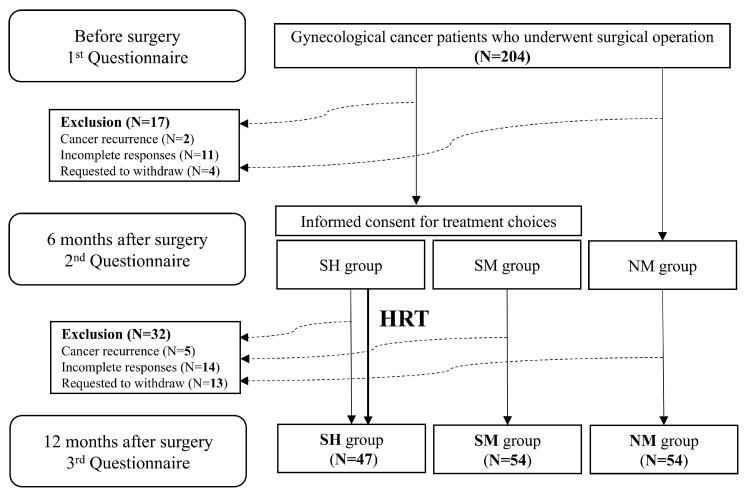
Flow chart summarizing the prospective cohort analysis of this study. Abbreviation: HRT, hormone replacement therapy; SH, gynecological cancer patients who received HRT after surgical menopause; SM, gynecological cancer patients who did not receive after surgical menopause; NM, gynecological cancer patients undergoing surgery after natural menopause.

A flowchart summarizing our study is shown in Figure 1. Exclusion criteria removed 49 patients from the study. The final number of participants was 155: SH (N = 47), SM (N = 54), and NM group (N = 54).

### 2.2. Background and Purpose

Baseline characteristics included age (years), body mass index (BMI: kg/m^2^), marital status, number of children, and smoking status. Medical characteristics included cancer type, clinical stage, surgical method, handling of lymph nodes, and postoperative adjuvant therapy. Gynecological cancers were clinically staged according to the International Federation of Gynecology and Obstetrics (FIGO) stage system.

In response to each patient’s request, HRT was started from 6 months after surgery in the SH group. HRT was administered using one of the following estrogen preparations: conjugated equine estrogen (0.625 mg daily, orally), 17-β estradiol transdermal patch (0.72 mg every 2 days), or 17-β estradiol transdermal gel (1.0 mg daily).

### 2.3. Questionnaires

Before surgery and at 6 and 12 months after surgery, we requested participants to complete questionnaires. The questionnaires included the Functional Assessment of Cancer Therapy-General (FACT-G) to assess QOL, the Center for Epidemiologic Studies Depression Scale (CES-D) to evaluate emotions, and the Endocrine Symptoms Subscale-19 (ESS-19) for menopausal symptoms. We examined one-year progression of FACT-G, ESS-19 score and the percentage of patients with CES-D scores (≥16 points).

### 2.4. Details of the Questionnaires

#### 2.4.1. QOL and Menopausal Symptom Assessment by FACT-G and ESS-19

QOL was evaluated using FACT-G, a cancer-specific health-related QOL scale. The assessment was developed by Cella et al. in 1993 and consists of 4 subscales: physical well-being (PWB), with 7 items; social well-being (SWB), with 7 items; emotional well-being, (EWB) with 6 items; and functional well-being (FWB), with 7 items, totaling 27 items [18]. Higher scores indicate better QOL. FACT-G includes several subscales developed for specific organs or symptoms related to cancer. We used the FACT-Endocrine Symptoms (FACT-ES) scale to assess menopausal symptoms associated with surgical menopause. FACT-ES is a QOL instrument for breast cancer patients undergoing hormonal therapy [19]. FACT-ES incorporates 19 items related to menopausal symptoms (ESS-19). During its development, ESS-19 was created by comparing patients with breast cancer to those without breast cancer, ensuring consistency. It can also be a useful indicator for evaluating menopausal symptoms associated with surgical menopause. FACT-G scores range from 0 to 108, and 4 subscales have the following score ranges: PWB 0–28, SWB 0–28, EWB 0–24, and FWB 0–28. ESS-19 includes 19 items. Scores of each item range 0–4, and then ESS-19 scores range 0–76. For example, vasomotor symptoms (VMSs) and arthralgia are assessed by ES1 and BMR1, respectively. Lower scores indicate more severe symptoms. Details of each domain, item, and score for FACT-G and FACT-ES are summarized in Supplementary Table S1. Minimally important differences (MIDs) of FACT-G are reported to be approximately 5 points [20]. Improved QOL was defined as an increase in FACT-G score of 5 points or more. Maintained QOL was defined as a change in FACT-G score of 4 points or less. The Japanese versions of the FACT-G and FACT-ES (including ESS-19) used in this study have been previously validated and shown to have good reliability and construct validity in Japanese cancer populations. The internal consistency reliability, as measured by Cronbach’s alpha, is excellent for the FACT-G (α = 0.89–0.92) and good to excellent for the FACT-ES endocrine symptom subscale (α = 0.83–0.90) [18,19]. Permission to use the Japanese versions of the FACT-G and FACT-ES was obtained from the Developer Functional Assessment of Chronic Illness Therapy (FACIT) measurement system.

#### 2.4.2. Assessment of Emotional Distress by CES-D

Emotional distress was assessed using CES-D [21], a widely used depression scale with adequate reliability and validity. It consists of 20 items, including 7 items related to depressive mood, 7 related to physical symptoms, 2 related to interpersonal relationships, and 4 inquiring about positive emotions. The scale assesses the number of days with symptoms in the past week, ranging from none, 1–2 days, 3–4 days, to 5 days or more. Scores range from 0–60, with higher scores indicating stronger depressive symptoms. The cutoff point is 16, which has been found to be valid in terms of reliability and validity in Japan. A CES-D score of 16 or above is considered to be depression, and a score of 16 or below is considered to be non-depression. The Japanese version of the CES-D has demonstrated good internal consistency, with Cronbach’s alpha coefficients ranging from 0.84 to 0.89 in prior studies [22].

### 2.5. Statistical Analysis

Baseline and medical characteristics of the three groups were analyzed using the Kruskal–Wallis test for age and BMI and Fisher’s exact test for other categories. As the FACT-G and ESS-19 scores were not normally distributed (shown by the Kolmogorov–Smirnov test), intra-group comparisons were performed using Friedman’s test. Comparisons of the percentage of depression (i.e., CES-D scores ≥ 16) were performed using Cochran’s Q test. If there were significant differences, subsequent comparisons between two groups were evaluated using the Holm test. Correlation between CES-D and FACT-G scores was assessed using Spearman’s test.

To examine the efficacy of HRT, we examined factors associated with improved or maintained QOL and emotion after 6 to 12 months in the SH and SM groups. Such factors were identified using univariate analysis through the chi-squared test, and multivariate logistic regression analysis. Independent variables included group (SH or SM), age, BMI, cancer type, FIGO stage, type of surgery, treatment of lymph node, adjuvant therapy, marital status, number of children, and smoking status. Based on the FIGO stage, early stage was classified as stage I, and late stage was classified as stage Ⅱ or more. Baseline variables with *p* < 0.30 in univariate analysis and clinically established important factors were included in the multivariate analysis. To evaluate the model fit and multicollinearity, we calculated the Akaike Information Criterion (AIC), performed the Hosmer–Lemeshow goodness-of-fit test, and assessed the variance inflation factor (VIF) for included variables. Statistical significance was set at *p* < 0.05. The EZR v.1.6 software (https://www.jichi.ac.jp/usr/hema/EZR/download.html, downloaded on 28 February 2022) was used [23].

## 3. Results

Table 1 shows the baseline and medical characteristics. The median age was different among the three groups (*p* < 0.001). The major cancer type in both SM and NM groups was endometrial cancer, while in the SH group, endometrial and cervical cancers were similarly prevalent. Lymphadenectomy was commonly performed in SH (*p* = 0.026), and the percentage of married women was higher in NM (*p* = 0.001) compared to the other two groups. In the SH group, the detailed methods of HRT administered 6 to 12 months after surgery included 17-βestradiol transdermal patch (0.72 mg, every 2 day) in 40 cases, 17-βestradiol transdermal gel (1.0 mg, daily) in 6 cases, and conjugated equine estrogen (0.625 mg, daily, orally) in 1 case (Supplementary Table S2). The amount of estradiol was determined to be equivalent between the routes of administration.

### 3.1. Changes in QOL, Emotional Distress, and Menopausal Symptoms

**Figure 2:**
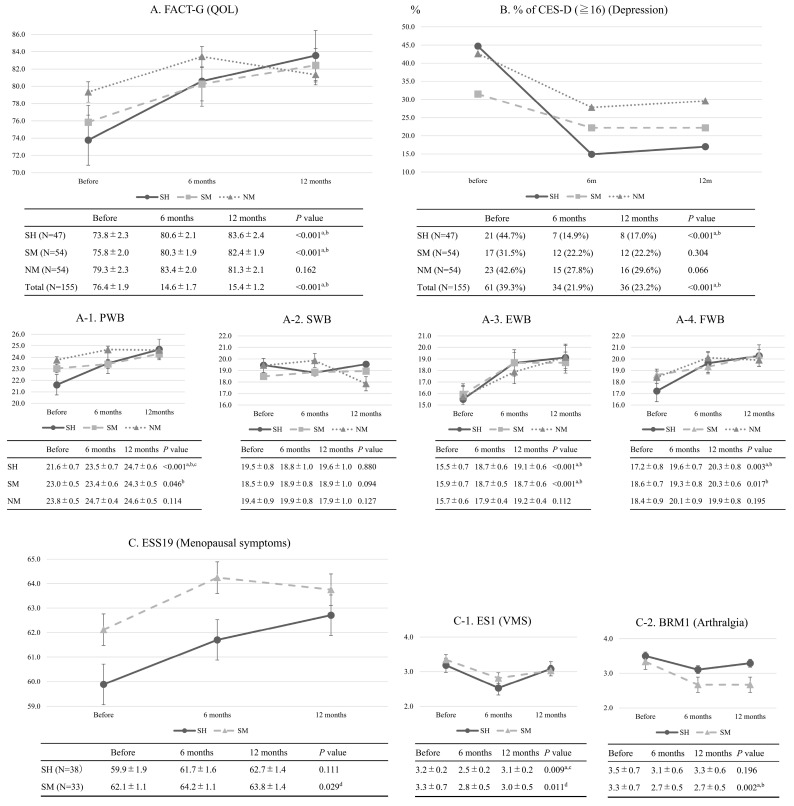
Outcomes of time-related changes in the FACT-G, CES-D, and ESS-19. Dates are expressed by mean ± standard error (SE) or numerical value (%). ^a^: Significant difference between before vs. 6 months, ^b^: Significant difference between before vs. 12 months, ^c^: Significant difference between 6 vs. 12 months, ^d^: Significant difference between the three groups. Abbreviation: SH, gynecological cancer patients who received HRT after surgical menopause; SM, gynecological cancer patients who did not receive after surgical menopause; NM, gynecological cancer patients undergoing surgery after natural menopause; FACT-G, Functional Assessment of Cancer Therapy-General; PWB, physical well-being; SWB, social well-being; EWB, emotional well-being; FWB, functional well-being; ESS-19, Endocrine Symptom Scale-19; CES-D, the Center for Epidemiologic Studies Depression Scales; QOL, quality of life; VMSs, vasomotor symptoms; HRT, hormone replacement therapy.

Figure 2 presents changes in FACT-G, its 4 subscales, prevalence of depression estimated by CES-D (≥16), ESS-19, and its 2 subscales. FACT-G scores were the lowest before surgery and gradually improved by 6 months after surgery in both SH and SM; they then remained stable for the next 6 months. Similar findings were observed in EWB and FWB changes. In NM, however, there were no significant changes in FACT-G and its subscales during the observation period. Although preoperative FACT-G scores were lowest in each group, SM and SH were 3 to 5 points lower than NM. Sexual activity could not be surveyed because of a high percentage of refusal to answer (47%).

We surveyed menopausal symptoms associated with surgical menopause using ESS-19 in SH (N = 38) and SM (N = 33). In intra-group comparison, there was no significant change in ESS-19 scores before and by 6 months after surgery, showing no worsening of overall menopausal symptoms after surgical menopause. Furthermore, there were no significant changes in menopausal symptoms between 6 and 12 months after surgery. However, worsened VMSs, estimated with ES1 at 6 months postoperatively, improved in SH, while worsened arthralgia, estimated with BRM1, persisted in SM.

In all participants (N = 155), depression rate was 39% (61/155) before surgery, this decreased to 21.9% (34/155, *p* < 0.01) by 6 months postoperatively, and remained unchanged at 23.2% (36/155, NS) for the next 6 months. In SH, similar changes in depression rate were observed, but in NM, the high depression rate did not improve during the observation period. Spearman’s rank correlation coefficients between CES-D and FACT-G scores were r = −0.606~0.637, *p* < 0.001 at the three time points, showing inverse correlations.

### 3.2. Examination of the Efficacy of HRT in GCSs After Surgical Menopause

Table 2 and Table 3 present the outcomes of uni- and multi-variate analysis to identify the factors influencing QOL and non-depression status from 6 to 12 months after surgery in SH and SM. Of all participants (N = 101), 81 cases (80.2%) improved or maintained QOL, and 81 cases (80.2%) maintained non-depression status. Baseline variables with *p* < 0.30 in univariate analysis were FIGO stage, marital status, and number of children. Marital status was excluded from multivariate analysis because of a potential confounding effect of having children. Multivariate analysis was performed with FIGO stage and number of children. The final model showed acceptable fit (AIC = 95.704, Hosmer–Lemeshow test *p* = 0.663), and no significant multicollinearity was observed (VIFs < 2). Parenthood was independently associated with maintained non-depression status. However, in both SH or SM, HRT was not correlated with changes in QOL and depression status.

## 4. Discussion

Only limited data are available with regard to the mental health and QOL of GCSs in Japan and other Asian countries. To our knowledge, this is the first prospective longitudinal study on the effects of surgical menopause and HRT on mental health, QOL, and menopausal symptoms in Japanese GCSs. We found that emotional distress, including anxiety and depression, and QOL changed during the cancer journey. The highest depression rate and the lowest QOL were observed at cancer disclosure. An inverse relationship between FACT-G and CES-D suggests that emotional distress leads to QOL decline, thus our findings agreed with previous reports [24,25,26]. Cancer disclosure affects mental health through evoking thoughts of death, resulting in severe stress [27]. Meanwhile, treatments such as anesthesia, surgery, and post-operative complications, fears of cancer recurrence, and stress from physical changes can lead to mood disturbances and depression. Furthermore, the loss of identity as a woman, along with fertility, and sexuality due to the removal of uterus and ovaries can also lead to emotional distress. These symptoms are a natural reaction and tend to improve over time. If depression and declined QOL persist and interfere with daily life, it is important to seek professional mental health care [28]. The rate of depression among patients before surgery is approximately 40%, which is consistent with findings from a previous study conducted in Japan [29]. Prevalence of depression at cancer disclosure in Japanese individuals is approximately 30–50%, which is about twice as high as the 15–25% observed in North America and Western Europe [30,31]. The depression rate in the general Japanese female population is reported to be 14.1% [32]. Although emotional distress and QOL improved by 6 months after surgery, 23% of cancer survivors showed persistent depression even one year after surgery. This percentage is also twice as high as the 10% found in a meta-analysis of the North America and Western Europe [31]. In summary, depression rates in Japanese GCSs seem to be twice as high as in North America and Western Europe before and one year after surgery. Another limitation of our study is that we did not stratify depression outcomes by age group. Age is a known factor influencing emotional vulnerability, particularly in the context of cancer and menopause. Therefore, future studies should investigate how age differences may affect psychological distress and QOL in this population.

The higher prevalence of depression in Japanese GCSs suggests that these patients continue to struggle with a fear of cancer recurrence and treatment complications, and therefore, do not normalize their mental health even one year postoperatively. Possible reasons for this may include the following. First, cultural characteristics of endurance and no expression of emotions in Japanese individuals may be responsible [29]. Second, Japanese have lower self-esteem, making them more prone to depression in response to cancer disclosure [33]. Third, cancer has been recognized as a curable disease in North America and Western Europe while in Japan, until recently, it has been perceived as an incurable disease with a terrifying image [34]. Finally, a delayed practice of cancer disclosure and survivorship in Japan may be responsible [35,36]. The emotional impact observed in our study must be interpreted within the context of Japanese cultural values and healthcare practices. In Japan, cancer disclosure practices have historically differed from Western approaches, with family members often being informed before patients, and disclosure being more gradual and indirect [37,38]. Although direct disclosure to patients has become more common in recent decades, the cultural emphasis on maintaining harmony (wa) and avoiding direct confrontation with distressing information may influence how Japanese women process and respond to cancer diagnosis. For broader applicability, future studies should include multi-national or cross-cultural cohorts.

At cancer disclosure, SM and SH were 3 to 5 points lower in FACT-G compared to NM. The median age of NM was late 50s, while that of SH and SM was 40s, of whom 50% or more had entered the menopausal transition. During this period, women are vulnerable to various types of physical and emotional distress [39]. Thus, the two surgical menopause groups may have felt a greater emotional distress in response to cancer disclosure. In addition, compared to NM, surgical menopause groups may have felt a greater loss of female identity, sexuality, and fertility, leading to greater emotional distress, as mentioned above.

Menopause is associated with morbidity of VMSs, anxiety, and depression [40]. Anxiety and depression also occur in GCSs. Menopausal HRT has been used to alleviate VMSs, genitourinary syndrome, anxiety, and depression [41]. However, it remains uncertain as to whether HRT has the potential to alleviate anxiety, depression, and declined QOL in GCSs who underwent surgical menopause [42,43]. In this observational study, QOL and depression outcomes appeared similar between patients who received HRT and those who did not. However, HRT was not randomly assigned, and patients who opted for HRT were significantly younger. Therefore, no causal conclusions regarding the efficacy of HRT on QOL or depression can be drawn from these results. However, having children was associated with persistence of a non-depression status, and these results agree with previous reports [44,45]. The relationship between hormone replacement therapy and cancer-related emotional distress operates through multiple interconnected pathways. From a neurobiological perspective, estrogen deficiency following surgical menopause can exacerbate mood disorders through several mechanisms: (1) disruption of serotonergic neurotransmission, which is closely linked to mood regulation; (2) alterations in the hypothalamic–pituitary–adrenal (HPA) axis, potentially amplifying stress responses to cancer diagnosis; and (3) changes in neuroplasticity and cognitive function that may affect coping mechanisms [46,47,48]. However, the theoretical framework becomes more complex when considering cancer-related psychological factors. The biopsychosocial model suggests that cancer-related emotional distress stems from multiple sources, including existential concerns, body image changes, fear of recurrence, and disruption of life goals—factors that may not be directly amenable to hormonal intervention. Therefore, while HRT may address the neurobiological components of mood disturbance related to estrogen deficiency, its efficacy for cancer-specific psychological distress may be limited [49,50]. We consider that GCSs experience both menopause- and cancer-related symptoms. VMSs and genitourinary syndrome mainly belong to the former. Depression and QOL that decline in response to cancer disclosure definitely belong to the latter. Meanwhile, QOL and depression decline after cancer surgery belong to both. It is likely that the impact of cancer-related distress is greater than that of menopause-related distress. Even though HRT alleviates menopause-related anxiety and depression, and these are related to QOL decline, the efficacy of HRT may be masked by the greater cancer-related emotional distress and QOL decline, thus making them incurable with HRT [44]. Indeed, a large-scale survey by Pearman et al. revealed that menopausal symptoms are not only hormone-dependent but are also influenced by psychological, social, and cultural factors, including overall health, mood, and life stressors [51]. Moreover, it is increasingly recognized that cancer survivors have a distinct physiological and cellular environment, characterized by chronic inflammation and alterations in hormone receptor expression due to cancer treatments, which may further influence their response to HRT [50,52]. These changes could potentially alter the expected therapeutic effects of HRT in alleviating depression or improving QOL in GCSs. These changes could potentially alter the expected therapeutic effects of HRT in alleviating depression or improving QOL in GCSs. In this study, HRT was not administered in a uniform way. Participants in the SH group chose from patches, gels, or oral tablets, all given at standard doses. While systemic estradiol exposure was assumed to be similar, the different routes may have caused slight variation. HRT duration was consistent, from six to twelve months postoperatively. Thus, we did not analyze outcomes by HRT type or duration. Still, this variability should be noted, and future studies should consider standardized protocols. In addition, HRT cannot always alleviate all menopausal symptoms [12,13,53]. Furthermore, for young gynecologic cancer patients scheduled to undergo surgical menopause, early implementation of fertility preservation strategies is essential, as the procedure results in irreversible loss of fertility. Adjunctive approaches such as nutraceuticals (e.g., myo-inositol, alpha-lipoic acid) may support reproductive function and metabolic balance before treatment-induced ovarian failure [54,55]. These compounds have shown promise in enhancing ovulatory function and stabilizing mood and may complement hormonal and psychological interventions. Oocyte vitrification is a critical preoperative option for preserving fertility among gynecologic and breast cancer patients and should be discussed proactively with eligible women [56]. In addition to physiological support, a gender-sensitive, couple-oriented psychological approach is crucial—particularly in addressing the emotional impact of infertility, identity loss, and relationship dynamics following surgical menopause [57]. Emotional distress related to infertility is not limited to female patients; partners, including men, may also experience significant psychological strain, which can influence overall family well-being. Moreover, molecular biomarkers such as circulating microRNAs (e.g., miR-125) are gaining attention for their potential in early detection and monitoring of hormone-sensitive cancers, particularly in fertility-preserving contexts. These may eventually help guide individualized survivorship and reproductive planning for patients undergoing abrupt hormonal changes due to cancer treatment [58,59]. Integrating these multidisciplinary strategies—metabolic, reproductive, psychological, and molecular—may significantly enhance the quality of survivorship care for young women undergoing surgical menopause. Future studies should explore these avenues to develop more comprehensive and personalized care models.

Finally, in this study, HRT was started based on each patient’s request. The SH group was significantly younger than the SM group; therefore, in the future, a randomized-controlled trial is absolutely necessary. However, the present study does not deny the efficacy of HRT, because recent evidence indicates that HRT ameliorates menopausal symptoms and bone loss, and that these are associated with QOL decline in GCSs [14]. Moreover, in this study HRT alleviated VMSs and did not aggravate arthralgia. However, to prevent or treat emotional distress and decline in QOL in GCSs, social and mental interventions and psychiatric support are primarily important [29].

Limitations of this study include the following. First, sample size was small. Thus, mental health and QOL could not be compared among the three types of gynecological cancers. These cancers have different etiology, treatment complications, and survival rates, leading to different impacts on mental health [60,61]. Second, we could not survey the effects of postoperative sexual dysfunction [62], complications [63], and other factors affecting mental health [64]. Third, HRT was not uniformly administered with a single agent but was prescribed variably depending on the patient’s request and medical condition. Therefore, the possibility of method bias due to variability in HRT modalities cannot be excluded. A future randomized–controlled trial delivering consistent HRT regimens is needed to better assess efficacy. Fourth, we were unable to clearly distinguish between the effects attributable to cancer diagnosis and treatment versus those specifically related to menopause. The participants in our surgical menopause groups (SH and SM) experienced both cancer-related stress and acute hormonal changes simultaneously, making it difficult to isolate the independent contribution of each factor to changes in QOL, depression, and menopausal symptoms. This confounding factor limits our ability to definitively attribute the observed improvements in FACT-G and CES-D scores solely to recovery from surgical menopause or to psychological adaptation following cancer treatment. Finally, this study covered only a one-year period, which is insufficient to survey mental health and QOL in cancer survivors. A long-term follow-up study is needed. In addition, factors influencing mental health and QOL in cancer patients are multidimensional [65,66]. The human mind and body are complex, and we are all individually different. Thus, factors influencing emotional distress and QOL might not be uniformly explained. Furthermore, socioeconomic factors such as occupation, education level, income, and hospital costs may also have significant impacts on mental health and QOL. These variables were not assessed in this study, and their influence remains unclear. Future studies should consider incorporating these factors to gain a more comprehensive understanding of the determinants of QOL in GCSs.

## 5. Conclusions

Based on these results, we concluded that the prevalence of depression was highest at cancer disclosure. It created greater psychological stress, leading to QOL decline. Declined QOL gradually improved by 6 months after surgery in surgical menopause groups but remained stable for the next 6 months. Although HRT is known to alleviate menopause-related VMSs, genitourinary syndrome, bone loss, and emotional distress, it does not seem to alleviate cancer-related emotional distress or improve QOL in the current study. Post-treatment QOL is associated with prolonged survival. Thus, interventions of mental, psychological, and social supports are important for patients after gynecological cancer surgery.

## Figures and Tables

**Table 1:** Characteristics of the three groups (N = 155).

Characteristics	SH (N = 47)	SM (N = 54)	NM (N = 54)	*p*-Value
Age (years)				
Median (range)	41 (27–53)	48 (28–56)	59 (50–73)	<0.001 *
<40	19 (40.4%)	5 (9.3%)	0	
40–49	26 (55.3%)	27 (50.0%)	0	
50–59	2 (4.3%)	22 (40.7%)	23 (42.6%)	
≥60	0	0	31 (57.4%)	
BMI (kg/m^2^)				
Median (range)	23.2 (16.5–48.1)	25.3 (16.8–51.3)	23.7 (17.2–38.8)	0.240
<25	32 (68.1%)	26 (48.1%)	31 (57.4%)	
25–30	7 (14.9%)	17 (31.5%)	12 (22.2%)	
>30	8 (17.0%)	11 (20.4%)	11 (20.4%)	
Cancer type and FIGO stage				0.006 *
Endometrial cancer	21 (44.7%)	40 (74.1%)	40 (74.1%)	
	0:1, I:17, II:1, III:1, IV:1	0:3, I:28, II:4, III 5, IV:0	0:0, I:33, II:3, III:2, IV:2	
Cervical cancer	22 (46.8%)	8 (14.8%)	8 (14.8%)	
	I:13, II:7, III:2, IV:0	I:4, II:4, III:0, IV:0	I:5, II:3, III:0, IV:0	
Ovarian cancer	5 (10.6%)	10 (18.5%)	7 (13.0%)	
	I:2, II:1, III:2, IV:0	I:6, II:2, III:2, IV:0	I:2, II:2, III:3, IV:0	
Type of surgery				
Laparoscopic or robotic surgery	16 (34.0%)	26 (48.1%)	30 (55.6%)	0.089
Laparotomy	31 (66.0%)	28 (51.9%)	24 (44.4%)	
Treatment of lymph node				
None or biopsy	14 (29.8%)	29 (53.7%)	29 (53.7%)	0.026 *
Lymph node dissection	33 (70.2%)	25 (46.3%)	25 (46.3%)	
Adjuvant therapy				
Chemotherapy	11 (23.4%)	21 (38.9%)	20 (37.0%)	0.141
Radiotherapy	12 (25.5%)	5 (9.3%)	6 (11.1%)	
None	24 (51.1%)	28 (51.9%)	28 (51.9%)	
Marital status				
Married	32 (68.1%)	32 (59.3%)	48 (88.9%)	0.001 *
Unmarried	15 (31.9%)	22 (40.7%)	6 (11.1%)	
Number of children				
One or more children	28 (59.6%)	33 (61.1%)	43 (79.6%)	0.053
No children	19 (40.4%)	21 (38.9%)	11 (20.4%)	
Smoking status				
Smoker	7 (14.9%)	9 (16.7%)	4 (7.4%)	0.314
Non-smoker	40 (85.1%)	45 (85.3%)	50 (92.6%)	

*: *p* < 0.05. Abbreviation: SH, gynecological cancer patients who received HRT after surgical menopause; SM, gynecological cancer patients who did not receive after surgical menopause; NM, gynecological cancer patients undergoing surgery after natural menopause; BMI, body mass index, FIGO, International Federation of Gynecology and Obstetrics; HRT, hormone replacement therapy.

**Table 2:** Uni-variate analysis to identify the factors influencing QOL and non-depression status in SH and SM groups (N = 101).

	Improved or Maintained QOL		Maintained Non-Depression Status	
	81 (80.2%)		81 (80.2%)	
Independent Variable	N	*p*-Value	N	*p*-Value
Group				
SH	37	0.805	39	0.619
SM	44		42	
Age				
<45 years	38	0.809	39	1.000
≥45 years	43		42	
BMI (kg/m^2^)				
<25	47	0.620	47	0.620
≥25	34		34	
Cancer type				
Endometrioid cancer	46		47	
Cervical cancer	23	1.000	23	0.649
Ovarian cancer	12		11	
FIGO stage				
Early stage	56	0.786	59	0.283
Late stage	25		22	
Type of surgery				
Laparoscopic or robotic surgery	35	0.616	33	0.802
Laparotomy	46		48	
Treatment of lymph node				
Lymph node dissection	36	0.614	46	1.000
None or biopsy	45		35	
Adjuvant therapy				
Chemotherapy or radiotherapy	40	0.805	38	0.620
None	41		43	
Marital status				
Married	52	0.798	54	0.199
Unmarried	29		27	
Number of children				
One or more children	47	0.445	55	0.004 *
No children	34		26	
Smoking status				
Smoker	14	0.732	12	0.516
Non-smoker	67		69	

Baseline variables with *p* < 0.30 in univariate analysis and clinically established important factors were included in the multivariate analysis. *: *p* < 0.05. Abbreviation: CI, confidence interval; SH, gynecological cancer patients who received HRT after surgical menopause; SM, gynecological cancer patients who did not receive after surgical menopause; BMI, body mass index; FIGO, International Federation of Gynecology and Obstetrics; HRT, hormone replacement therapy.

**Table 3:** Multi-variate analysis to identify the factors of non-depression status in SH and SM groups (N = 101).

Independent Variable	Odds Ratio (95%CI)	*p*-Value
FIGO stage		
Early stage	1.89 (0.641–5.59)	0.248
Late stage	1 (ref)	
Number of children		
One or more children	5.04 (1.72–14.8)	0.003 *
No children	1 (ref)	

Multivariate analysis was performed with FIGO stage and number of children unconfounded by baseline variables that had *p* < 0.30 in univariate analysis. Model fit was confirmed using AIC (95.704) and the Hosmer–Lemeshow test (*p* = 0.663). Multicollinearity was not observed; all VIF values were <2. *: *p* < 0.05. Abbreviation: CI; Confidence interval, FIGO; International Federation of Gynecology and Obstetrics.

## Data Availability

The data for this study are shown in tables and figures no other datasets were generated or analyzed during the current study.

## Supplementary Materials

The following supporting information can be downloaded at: https://www.mdpi.com/article/10.3390/medicina61071191/s1, Table S1: Details of each domain of FACT-G and FACT-ES, Table S2: Details of the HRT method used by the SM group (N = 47) from 6 to 12 months after surgery.
