# Supplementary material for: Changes in Quality of Life, Depression, and Menopausal Symptoms After Surgical Menopause and the Efficacy of Hormone Replacement Therapy in Gynecological Cancer Survivors: A One-Year Prospective Longitudinal Study
Source: Medicina (Kaunas). 2025 Jun 30;61(7):1191. doi: 10.3390/medicina61071191 (PMC12299879; doi:10.3390/medicina61071191)
Supplement: Supplementary file 1 [file medicina-61-01191-s001.zip › medicina-3709220-supplementary.pdf]

Supplementary table1. Details of each domain of FACT-G and FACT-ES

		Category	Number of Items	Items	Score range	The cut-off value of low QOL score
FACT-ES	FACT-G	PWB (Physical Well-Being)	7 items	GP1-7	0-28	15
		SWB (Social Well-Being)	7 items	GS1-7	0-28	16.7
		EWB (Emotional Well-Being)	6 items	GE1-6	0-24	13.5
		FWB (Functional Well-Being)	7 items	GF1-7	0-28	11.6
		ESS-19 (Endocrine Symptoms Subscale)	19 items	ES1-13, An9, O2, C2, An10, Tax1, BRM1	0-76	-

Abbreviation: FACT-G; the Functional Assessment of Cancer Therapy-General, FACT-ES; the Functional Assessment of Cancer Therapy-Endocrine Symptoms. Items are internal identifiers, not abbreviation.

Supplementary table2. Details of the HRT method used by the SM group (N=47) from 6 to 12 months after surgery.

Medications	SH (N=47)
17-β estradiol transdermal patch (0.72mg, every 2 day)	40
17-β estradiol transdermal gel (1.0mg, daily)	6
conjugated equine estrogen (0.625mg, daily, orally)	1

Abbreviation: SH; Gynecological cancer patients who received HRT after surgical menopause, HRT; Hormone replacement therapy.
